# Crude aqueous extracts of *Pluchea indica* (L.) Less. inhibit proliferation and migration of cancer cells through induction of p53-dependent cell death

**DOI:** 10.1186/1472-6882-12-265

**Published:** 2012-12-26

**Authors:** Jonathan J Cho, Chung-Lung Cho, Chiu-Li Kao, Chien-Ming Chen, Chao-Neng Tseng, Ya-Zhe Lee, Li-Jen Liao, Yi-Ren Hong

**Affiliations:** 1 Department of Biology, Rensselaer Polytechnic Institute, Troy, NY, 12180, USA; 2 Department of Biological Sciences, National Sun Yat-sen University, Kaohsiung, Taiwan; 3 Tzu Hui Institute of Technology, Pingtung County, Taiwan; 4 Graduate Institute of Natural Products, Kaohsiung Medical University, Kaohsiung, Taiwan; 5 Department of Biotechnology, National Kaohsiung Normal University, Kaohsiung, Taiwan; 6 Department of Biochemistry, Faculty of Medicine, Kaohsiung Medical University, Kaohsiung, Taiwan

**Keywords:** *Pluchea indica* (L.) Less, Crude aqueous extracts, Cancer, p53, p21, Cell growth arrest, Apoptosis

## Abstract

**Background:**

*Pluchea indica* (L.) Less. (Asteraceae) is a perennial shrub plant with anti-inflammatory and antioxidant medicinal properties. However, the anti-cancer properties of its aqueous extracts have not been studied. The aim of this study was to investigate the anti-proliferation, anti-migration, and pro-apoptotic properties of crude aqueous extracts of *P. indica* leaf and root on human malignant glioma cancer cells and human cervical cancer cells, and the underlying molecular mechanism.

**Methods:**

GBM8401 human glioma cells and HeLa cervical carcinoma cells were treated with various concentrations of crude aqueous extracts of *P. indica* leaf and root and cancer cell proliferation and viability were measured by cell growth curves, trypan blue exclusions, and the tetrazolium reduction assay. Effects of the crude aqueous extracts on focus formation, migration, and apoptosis of cancer cells were studied as well. The molecular mechanism that contributed to the anti-cancer activities of crude aqueous extracts of *P. indica* root was also examined using Western blotting analysis.

**Results:**

Crude aqueous extracts of *P. indica* leaf and root suppressed proliferation, viability, and migration of GBM8401 and HeLa cells. Treatment with crude aqueous extracts of *P. indica* leaf and root for 48 hours resulted in a significant 75% and 70% inhibition on proliferation and viability of GBM8401 and HeLa cancer cells, respectively. Crude aqueous extracts of *P. indica* root inhibited focus formation and promoted apoptosis of HeLa cells. It was found that phosphorylated-p53 and p21 were induced in GBM8401 and HeLa cells treated with crude aqueous extracts of *P. indica* root. Expression of phosphorylated-AKT was decreased in HeLa cells treated with crude aqueous extracts of *P. indica* root.

**Conclusion:**

The *in vitro* anti-cancer effects of crude aqueous extracts of *P. indica* leaf and root indicate that it has sufficient potential to warrant further examination and development as a new anti-cancer agent.

## Background

Cancer is a prevalent medical problem with high mortality and wide epidemiology [1,2]. It is a complex disease caused by numerous factors ranging from external environmental chemicals to hereditary genetics. Current treatment of cancer can be categorized into three groups: surgery, chemotherapy, and radiation therapy [3-5]. Surgical removal of tumor and cancerous growth can effectively treat 50% of cancer but cannot eliminate all cancer cells, which results in high recurrence rate [4]. Chemotherapy and radiation therapy have the potential of removing all cancer cells but both lack the specificity to limit their damaging effect on cancer cells, thus harming normal body cells in the process and causing countless side-effects. Many chemotherapy chemicals target proliferating and high-metabolic cells, prominent characteristics of cancer cell and other fast-dividing cells, such as those of the integumentary system. The focal point of cancer research is still the search for effective and specific anti-cancer treatments [6].

The use of complementary and alternative therapies for cancer patients have grown in developed countries [6,7]. A recent study estimated the overall prevalence for the use of herbal products to be 13% to 63% among cancer patients [7]. A systematic review found that hypnosis, imagery, support groups, acupuncture, and healing touch seemed promising in relieving cancer pain in the short term; however, no formal recommendation can be made because of a paucity of rigorous randomized controlled trials [8]. In a study on the use of complementary and alternative medicine (CAM) by breast cancer survivors in Ontario, Canada, the investigators found that 66.7% of the respondents in a randomized survey reported using CAM [9]. The study concluded that CAM use is common among Canadian breast cancer survivors and that many are discussing CAM therapy options with their physicians [9]. A meta-analysis of randomized trials for combined *Astragalus*-based herbs and platinum-based chemotherapy, as compared to platinum-based chemotherapy alone, for advanced non-small-cell lung cancer found that *Astragalus*-based herbal medicine may increase effectiveness of platinum-based chemotherapy; however, rigorous controlled trials are required to confirm these results [10]. Thus, herbal medicine is emerging to play an important role in cancer treatment and survivor therapies.

*Pluchea indica* (Asteraceae) is a perennial shrub plant indigenous to many Asian countries [11]. Anti-inflammatory properties were found in the methanolic fraction of the chloroform extract of *P. indica* root [12]. The methanolic extract of *P. indica* root was found to possess the ability to neutralize viper venom and counter venom-induced lethality and hemorrhagic activity [13]. Significant anti-ulcer and anti-tuberculosis activities were found in the methanol fraction of *P. indica* root and leaf extracts, respectively [14,15].

This is the first study to demonstrate the *in vitro* anti-cancer property of *P. indica* aqueous extracts in the inhibition of cancer cell proliferation, focus formation, and migration. It was demonstrated that crude aqueous extracts of *P. indica* root effectively inhibited cancer cell proliferation, focus formation, and migration at low concentrations (100–300 μg/ml). Additionally, the underlying mechanism of anti-cancer activities may be attributed to the induction of critical tumor suppressor molecules, phosphorylated-p53 and p21, and decreased expression of an important survival signaling molecule, phosphorylated-AKT, in GBM8401 and HeLa cancer cells treated with crude aqueous extracts of *P. indica* root.

## Methods

### Cell culture

Human brain malignant glioma cell line GBM8401, and human cervical carcinoma cell line HeLa were used. GBM8401 was cultured in RPMI1640 (Invitrogen). HeLa was cultured in Dulbecco’s modified Eagle’s medium (DMEM). Both RPMI1640 and DMEM were made into complete medium with 10% cosmic calf serum (CCS; Hyclone), 200 mM L-glutamine (Invitrogen), 1% Penicillin (10000 units/ml)/Streptomycin (10000 units/ml) (Hyclone), 1X non-essential amino acids (NEAA; Invitrogen), 1 mM sodium pyruvate (Invitrogen). Cells were incubated at 37°C, in a humidified incubator with 5% CO_2_.

### Preparation of *Pluchea indica* crude aqueous extracts

Live plant material of *P. indica* was collected from the Yanchiao campus of National Kaohsiung Normal University, Kaohsiung, Taiwan. The plant material was authenticated and prepared as root and leaf dry powder by Professor L J Liao. The extraction of crude aqueous extracts of *P. indica* leaf or root was prepared by the method of Li et al. [16]. Briefly, 25 g of leaf or root powder were extracted three times in 200 ml of ddH_2_O (75°C-80°C) on a shaker. The resultant aqueous solution, which was filtered through 0.45-μm filters, was further concentrated by quickly frozen at −40°C and dried for 48 h using a freeze dryer (Savant Refrigerated vapor Trap, RV T41404, USA) to give a yield of 2.2 g crude leaf extracts (12.5%) and 1.3 g of crude root extracts (9.9%), respectively. The soluble aqueous extracts were dissolved in ddH_2_O to obtain stock solutions of *P. indica* leaf or root aqueous extract. Different desired concentrations of crude aqueous extracts were prepared by reconstitution with ddH_2_O and used in this study.

### Preliminary phytochemical screening of *Pluchea indica* crude aqueous extracts

The *P. indica* dry crude extract was evaluated for total phenol and flavonoids contents using Folin-Ciocalteu reagent and the aluminum chloride colorimetric method and was calculated as gallic acid equivalents in mg/g of extract and catechin equivalents in mg/g of extract, respectively, according to methods described previously with minor modifications [17]. Proanthocyanidins content was determined using the vanillin assay and expressed as catechin equivalents in mg/g of extract as described by Butler et al. [18]. All assays were carried out in triplicate.

### Determination of lipid peroxidation

Lipid peroxidation was measured in HeLa cells using the thiobarbituric acid (TBA) assay for malondialdehyde and normalized to total protein [19]. Briefly, cell pellets were mixed with 0.3% deaerated thiobarbituric acid solution in 3.9% (w/v) trichloroacetic acid and incubated at 95–100°C for 20 min. After cooling, the reaction mixtures were centrifuged at 10,000 rpm for 10 min. The supernatants were then loaded in 96-well plates and determined at an absorbance of 532nm/600nm. The MDA contents were calculated based on A_532_-A_600_ with the extinction coefficient of 155 mM^-1^ cm^-1^.

### Cell proliferation and viability assays

Trypan blue exclusions and the tetrazolium (3-(4,5-dimethylthiazol-2-yl)-5-(3-carboxymethoxyphenyl)-2-(4-sulfophenyl)-2H-tetrazolium; MTS) reduction assay were used to monitor the proliferation and viability of cancer cells upon exposure to different concentrations of aqueous extracts for different incubation periods. For the MTS assay, CellTiter 96 Aqueous One Solution Cell Proliferation Assay Kit (Promega, USA) was used by following manufacturer’s protocol. Briefly, 2 × 10^3^ cells/well was seeded in 96-well plates. Following a 24-hour incubation, *P. indica* leaf or root aqueous extract at 0, 100, 500, and 1000 μg/ml concentrations was added and incubated for 72 hours. After the 72-hour incubation, the medium was removed, 100 μl of medium with 20 μl of MTS solution was added, and the cells were incubated for 3 hours. Absorbance was read at 490 nm with an ELISA Reader (Micro Quant, BIO-TEK, USA) and analyzed using the KC4 3.3 Rev 10 program (BIO-TEK instruments Inc., USA). Trypan blue dye exclusion was used to assess the viability of cancer cells grown under different conditions as described above. Growth curves, each covering a total of 6 days of culturing, were constructed from which the effects of *P. indica* aqueous extract on population doubling time and saturation density were determined.

### Focus formation assay

One hundred cells were seeded in 60-mm plates and incubated at 37°C, 5% CO_2_ for 24 hours. The plates were prepared in triplicates. Following a 24-hour incubation, various concentrations of *P. indica* root aqueous extract were added. After 10 days, colonies were stained with crystal violet and counted.

### *In vitro “*scratch” wound closure assay

*In vitro* wound closure assay was performed as described previously [20]. In brief, HeLa cells with 90% confluence were wounded by scraping across the cell monolayer with a pipette tip. After washing with PBS, cells were incubated in the media supplemented with aqueous extracts of *P. indica* root at 0, 250, and 500 μg/ml concentrations, respectively. “Scratch” wound closure of the scratched surface was observed and photographed at 0-, 24-, 48-, and 72-hour time intervals.

### Apoptosis assay and flow cytometry analysis

Determination of apoptosis was conducted following manufacturer’s protocol by staining with FITC Annexin V Apoptosis Detection Kit I (BD Pharmingen, BD Biosciences, USA). Briefly, cells were plated in 100-mm culture dishes and allowed to grow to 70-80% confluence. They were treated with appropriate concentrations of aqueous extract and incubated for 2 days. The cells (1 × 10^5^cells/100 μl were collected and 5 μl of FITC Annexin V and 5 μl of propidium iodide (PI) were added. Cells were then incubated in the dark at room temperature. After 15 min, 400 μl of 1 × binding buffer was added to the cells. Flow cytometry analysis was conducted using BD LSR II Flow Cytometer (BD). Flow cytometry data were analyzed using BD FACSDiva software (BD).

### Western blot analysis

After treatment with *P. indica* aqueous extracts, cells were lysed with T-PER protein extraction reagent (Pierce), and 20 μg protein was used for immunoblotting analysis. Immunoblotting proteins were eluted by boiling for 5 min in a 1 × SDS sample buffer containing 5% (v/v) 2-mercaptoethanol. Proteins were resolved in a 12% SDS-PAGE and transferred to nitrocellulose membranes. The nitrocellulose membranes were immunoblotted with specific antibodies against p21 (Santa Cruz Biotechnology), phosphorylated-p53 (pS15) (Cell Signaling Technology), AKT (Cell Signaling Technology), phosphorylated-AKT (Cell Signaling Technology), Cleaved Caspase-3 (Asp175) (5A1E) Rabbit mAb (Cell Signaling Technology), α-tubulin (NeoMarkers), or β-actin (SIGMA), respectively. For visualization of immunoreactive bands, ECL+ reagents (Amersham) followed by chemiluminescence were used. Control and experimental groups were generated from the same blot.

### Statistical analysis

Data are presented as mean ± standard deviations (SD) of three independent experiments and statistical significance was determined using Independent Student’s *t*-test and the SPSS 12.0 software (SPSS Inc., USA). A significant difference was considered if *P* < 0.05.

## Results

### Phytochemical screening

The preliminary phytochemical analysis performed on crude aqueous extracts of *P. indica* leaf and root revealed the presence of tannins, saponins, flavonoids and proanthocyanidins. The total phenol content of the crude aqueous extract of *P. indica* root was 78.9 mg gallic acid equivalent/g of dry extract with reference to standard curve (y = 3.5547x - 0.0159, R^2^ = 0.9997) (Table 1). The total flavonoids and proanthocyanidins contents of the plant were 40.4 mg and 4.248 mg catechin equivalent/g of dry extract with reference to standard curves (y = 0.0038x −0.0075, R^2^ =0.9997 and y = 0.0118x + 0.0347, R^2^ = 0.9924), respectively (Table 1). The screened phytochemical compounds are known to support bioactive activities in medicinal plants and thus responsible for the anti-proliferation and anti-migration activities of the crude aqueous extracts of *P. indica* used in this study.

**Table 1:** **Phytochemical compounds of *Pluchea indica *root aqueous extract.**

**Phytochemical compounds**	**Amount present**	**Mean**^**N **^**± SD**	**Percentage in extract**
Flavonoids	++	ND	ND
Tannins	++	ND	ND
Saponins	+	ND	ND
Total phenol	+++	78.9±0.6 mg GAE/g DW	7.89
Total flavonoids	++	40.4±0.3 mg CE/g DW	4.04
Proanthocyanidins	+	4.248±0.068 mg CE/g DW	0.42

N=3; +++ = appreciable amount (positive within 3 min.); ++ = moderate amount (positive after 3 min., but within 5 min.); + = trace amount (positive after 5 min.); GAE = gallic acid equivalents; CE = catechin equivalents.

### Membrane lipid oxidation

**Figure 1:**
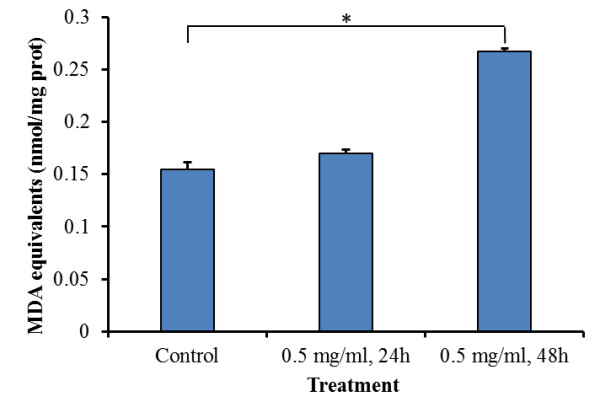
**Pro-oxidant activity of crude aqueous extract of *P. indica *root in HeLa cancer cells.** Measurement of thiobarbituric acid reactive species (TBARS) during malondialdehyde peroxidation in HeLa cells treated with 0.5 mg/ml of crude aqueous extracts at 24-hr and 48-hr incubation respectively were performed as described in Methods. Each experiment was done in triplicate. The pro-oxidant activity of the crude aqueous extract of *P. indica* root was evident. Data are expressed as mean ± SD. *P < 0.01.

The levels of lipid peroxidation (thiobarbituric acid reactive species; TBARS) was indirectly measured by monitoring the production of MDA equivalents from the oxidation of malondialdehyde in HeLa cells treated with 0.5 mg/ml of crude aqueous extracts of *P. indica* root after 24-hr and 48-hr incubation (Figure 1). A significant increase in MDA equivalents, which corresponds to an increase of lipid peroxide on membranes, was observed for cells incubated for 48-hr with crude aqueous extract. The pro-oxidant activity of the crude aqueous extracts of *P. indica* root in HeLa cancer cells was evident (Figure 1).

### Crude aqueous extracts of *P. indica* leaf and root suppress cancer cell growth

**Figure 2:**
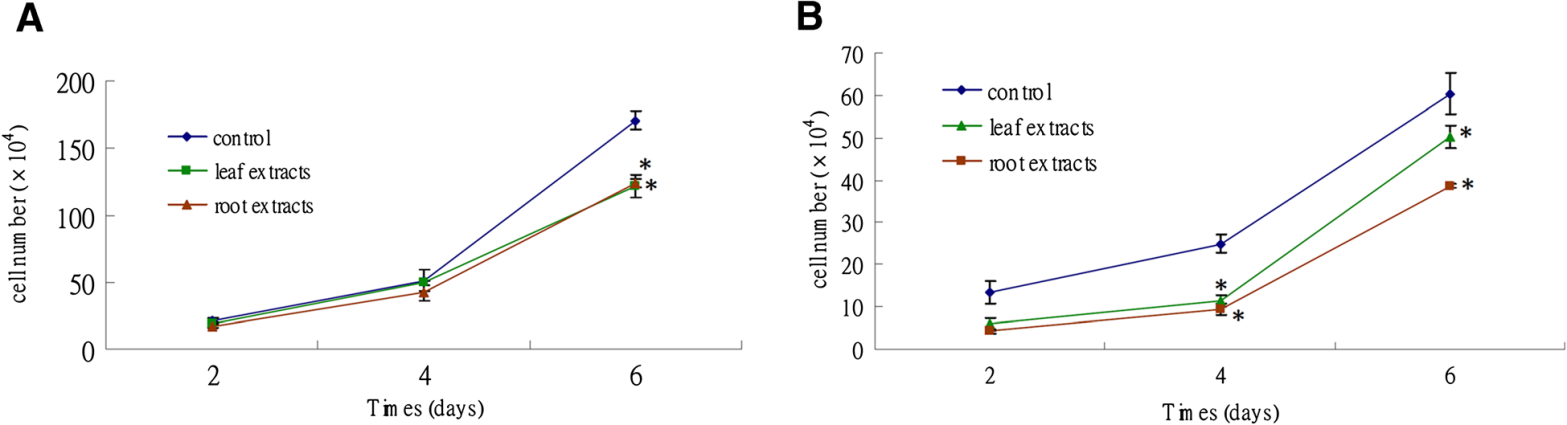
**Effect of *P. indica *leaf or root crude aqueous extract on the growth of cancer cells.** Growth curves of (**A**) GBM8401 cells and (**B**) HeLa cells after 300 μg/ml *P. indica* leaf or root aqueous extract treatment. Each experiment was done in triplicate. Data are expressed as mean ± SD. *P < 0.05.

High rate of proliferation is a hallmark of cancer cells. To examine the effect of *P. indica* leaf and root aqueous extracts on cancer cell proliferation, growth curve experiments were performed. The inhibitory effect of the extract treatment was time-dependent (Figure 2). It was observed that 300 μg/ml of *P. indica* leaf and root aqueous extracts suppressed GBM8401 brain cancer cell growth at day 6 as compared to untreated cells (*P* < 0.05) (Figure 2A). At both day 4 and day 6, the same dosage (300 μg/ml) of *P. indica* leaf and root aqueous extracts inhibited HeLa cervical cancer cell growth as compared to untreated cells (*P* < 0.05) (Figure 2B).

### Crude aqueous extracts of *P. indica* leaf and root decrease proliferation and viability of cancer cells

**Figure 3:**
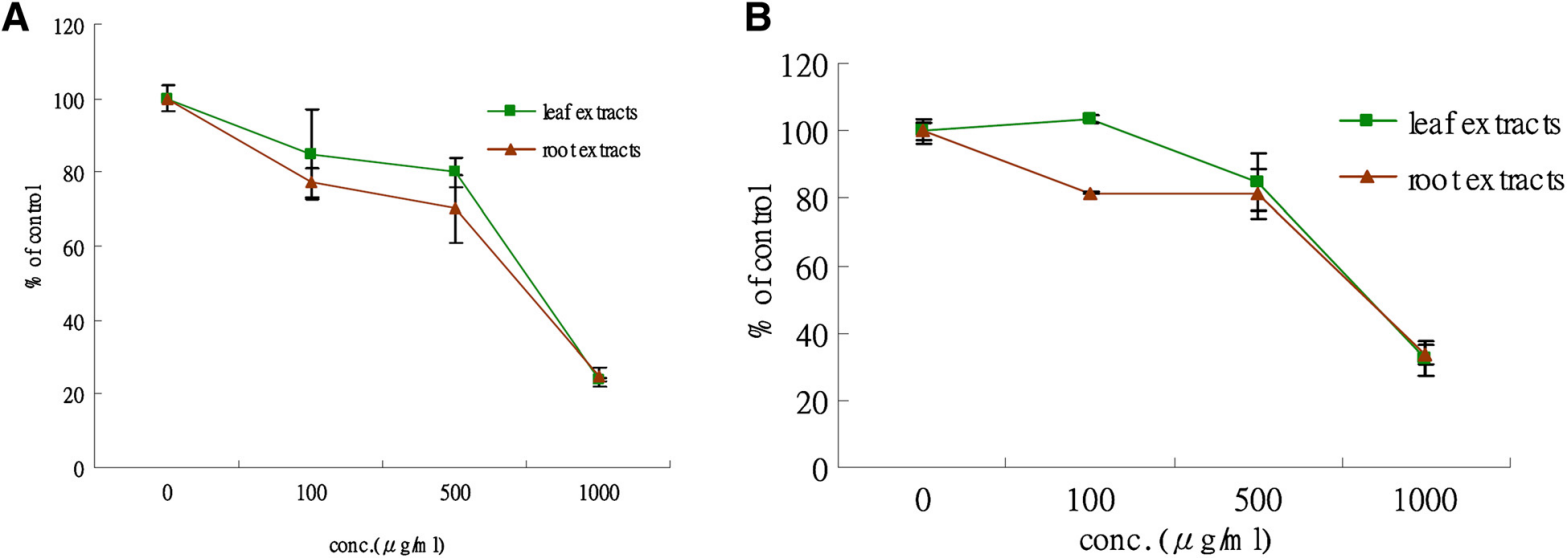
**Effect of *P. indica *leaf or root crude aqueous extract on cell proliferation and viability of cancer cells as determined by MTS assay.** MTS assay of (**A**) GBM8401 cells and (**B**) HeLa cells after 0, 100, 500, and 1000 μg/ml *P. indica* leaf or root aqueous extract treatment. Each experiment was done in triplicate. Data are expressed as mean ± SD.

To further determine the effect of *P. indica* leaf or root aqueous extracts on cancer cell proliferation and viability, MTS assay was performed. The effect of inhibition by these extracts was dose-dependent on both types of cancer cells (Figure 3). At the 1 mg/ml dose, both leaf and root extracts were effective, and treatment with 1 mg/ml for 72 hours resulted in 75% inhibition on GBM8401 brain cancer cells (Figure 3A). As shown in Figure 2B, treatment with leaf or root extract at the 1 mg/ml dose also resulted in significant inhibition (70%) of proliferation and viability of HeLa cells. The results indicated that *P. indica* aqueous extracts exerted anti-proliferation activity on GBM8401 and HeLa cancer cells.

### Crude aqueous extracts of *P. indica* root inhibits cancer cell focus formation

**Figure 4:**
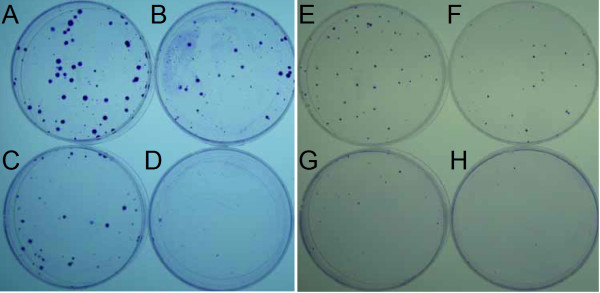
**Effect of *P. indica *root crude aqueous extract on the focus formation ability of cancer cells.** Focus formation assay of (**A**, **B**, **C**, **D**) GBM8401 cells and (**E**, **F**, **G**, **H**) HeLa cells after *P. indica* root aqueous extract treatment. (**A**, **E**) 0 μg/ml *P. indica* root aqueous extract treatment. (**B**, **F**) 10 μg/ml *P. indica* root aqueous extract treatment. (**C**, **G**) 100 μg/ml *P. indica* root aqueous extract treatment. (**D**, **H**) 200 μg/ml *P. indica* root aqueous extract treatment. Each experiment was repeated five times.

Focus formation assay was performed to examine the inhibitory effect of *P. indica* root aqueous extract on proliferation of cancer cells. Ten days after incubation in 10 μg/ml and 100 μg/ml *P. indica* root aqueous extracts, GBM8401 cells showed diminished focus formation, and HeLa cells showed marked decrease in focus formation after 100 μg/ml *P. indica* root aqueous extract treatment compared to untreated cells in both cell lines (Figure 4). When treated with 200 μg/ml *P. indica* root aqueous extract, both GBM8401 and HeLa cells showed nearly zero focus formation when compared to untreated cells (Figure 4D,H).

### Crude aqueous extracts of *P. indica* leaf and root represses cancer cell migration

**Figure 5:**
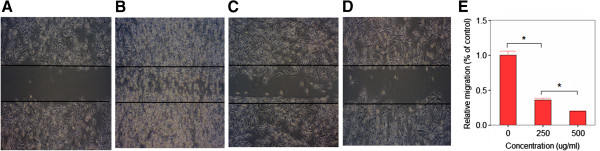
**Effect of *P. indica *root crude aqueous extract on migration of HeLa cells as determined by *in vitro *“scratch” wounjd closure assay.** “Scratch” wound closure assay of (**A**) HeLa cells after 0 hours. (**B**) HeLa cells after 48 hours incubation with 0 μg/ml *P. indica* root aqueous extract. (**C**) HeLa cells after 48 hours incubation with 250 μg/ml *P. indica* root aqueous extract. (**D**) HeLa cells after 48 hours incubation with 500 μg/ml *P. indica* root aqueous extract. (**E**) Statistical analysis of HeLa cells after 48 hours incubation with 0, 250, or 500 μg/ml *P. indica* root aqueous extract. Each experiment was done in triplicate. Error bars show mean ± SD; bars show means; *P < 0.05.

Many cancer cells exhibit migratory properties, and migration is essential for cancer cells during metastasis. *In vitro* “scratch” wound closure assay was performed to further examine the inhibitory effect of *P. indica* leaf or root aqueous extracts on the migratory activity of cancer cells. When treated with 250 μg/ml or 500 μg/ml leaf or root aqueous extracts and 250 μg/ml root aqueous extract, less migratory GBM8401 cells were found in the gap after 72 hours of extract incubation (data not shown). For HeLa cells, compared to control, less than 50% and 25% migrating cells can be found in the gap after 48 hours incubation with 250 μg/ml and 500 μg/ml *P. indica* root aqueous extract, respectively (Figure 5B,C,D,E). Thus, *P. indica* leaf and root aqueous extract, at concentrations of 250 μg/ml and 500 μg/ml, showed highly repressive effect on migration of HeLa cells.

### *P. indica* root aqueous extracts induce apoptosis in HeLa cervical carcinoma cells

**Figure 6:**
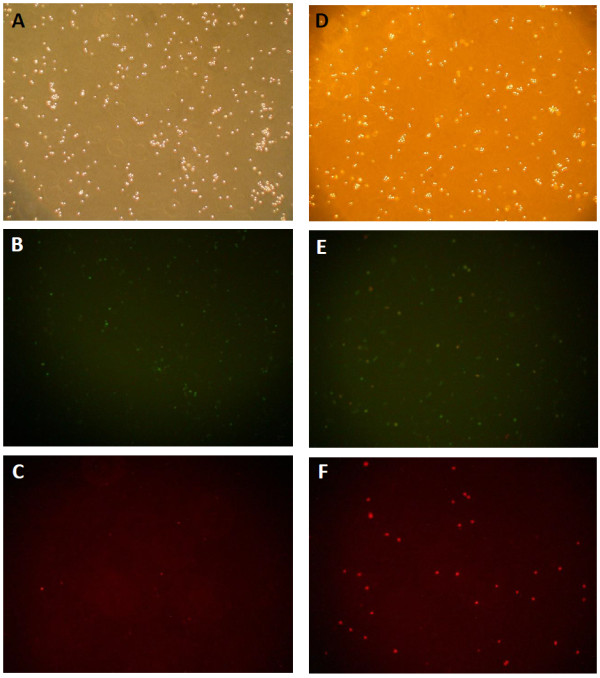
**Fluorescence microscopy of HeLa cells treated with crude aqueous root extracts of *P. indica *for 48 hours and double stained with annexin-V-sensitive probe (conjugated to FITC) and propidium iodide.** Qualitative labeling of annexin V in the plasma membrane (**B** and **E**) or cellular uptake of propidium iodide (**C** and **F**) was recorded. Control cells exposed to PBS (**A**)-(**C**), and 0.5 mg/ml of crude aqueous extracts treated cells (**D**)-(**F**) were photographed with a microscope (× 40).

**Figure 7:**
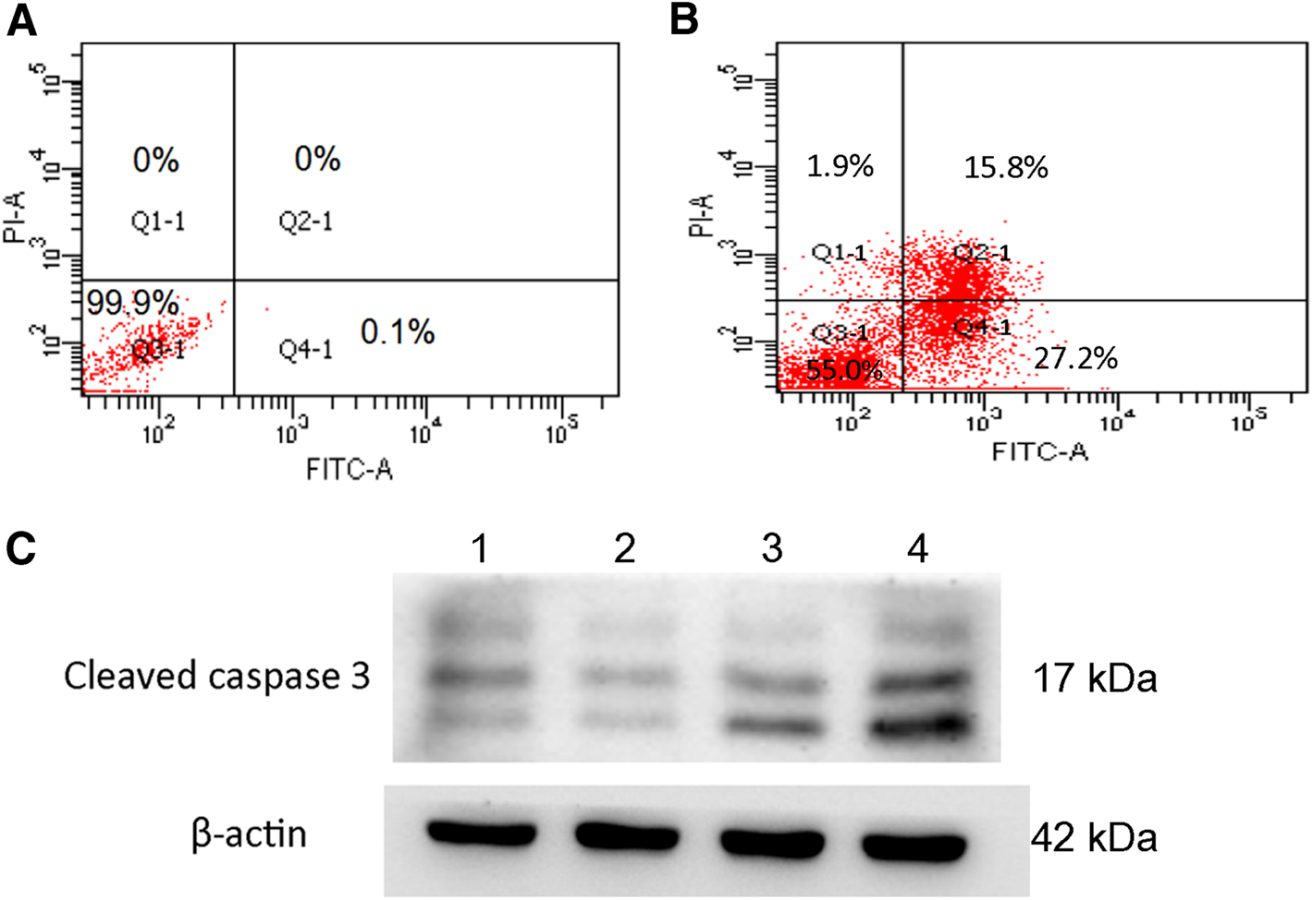
**Flow cytometry analysis of HeLa cells stained with FITC-Annexin V and PI and Western blotting of enhanced expression of cleaved caspase-3.** Flow cytometry analysis of FITC-Annexin V and PI stained HeLa cells with untreated HeLa cells (**A**) and HeLa cells treated with 0.5 mg/ml *P. indica* root extract for 48 hours (**B**). In each scatter plot, upper-left quadrant (Q1-1) shows naked nucleus cell mass, upper-right quadrant (Q2-1) shows necrotic cell mass, lower-left quadrant (Q3-1) shows survival cell mass, and lower-right (Q4-1) shows apoptotic cell mass. In control cells, there were only 0.1% of apoptotic cells (Figure 7A; Q4-1). After 48 hours of treatment with 0.5 mg/ml *P. indica* root aqueous extract, approximately 43% of cells were detected as undergoing necrosis and apoptosis (Figure 7B; Q2-1+Q4-1). (**C**) A representative of Western blotting of time-dependent increasing expression of cleaved caspase-3. Cleaved Caspase-3 (Asp175) monoclonal antibody (5A1E; Cell Signaling Technology) used detects levels of the large fragment (17/19 kDa) of activated caspase-3 resulting from cleavage adjacent to Asp175. Lane 1: untreated; lane 2: 1 day of 0.5 mg/ml extract treatment; lane 3: 2 days of 0.5 mg/ml extract treatment; lane 4: 3 days of 0.5 mg/ml extract treatment. Each experiment was done in triplicate.

The induction of apoptosis in cancer cells is another characteristic of anti-cancer agents. FITC-annexin V and propidium iodide (PI) staining assay was performed to examine the pro-apoptotic potential of *P. indica* root aqueous extract in HeLa cells. Fluorescence microscopy analysis (Figure 6) demonstrated that significant apoptosis occurred in HeLa cells treated with crude aqueous root extracts of *P. indica* for 48 hours as depicted by stronger staining of the cells by the Annexin V antibody (green; Figure 6E) as compared to the control cells (Figure 6B). PI staining (red; Figure 6F) was also noted, indicating late apoptotic or necrotic cells. To measure the percentage distribution of *P. indica* extract-induced apoptotic and necrotic HeLa cancer cells, flow cytometry was performed (Figure 7A and Figure 7B). In control HeLa cells, there was only 0.1% of apoptotic cells (Figure 7A; Quadrant 4–1). After 48 hours of treatment with 0.5 mg/ml *P. indica* root aqueous extract, approximately 43% of HeLa cells were detected as undergoing the end stage of necrosis and apoptosis (Figure 7B; Quadrant 2-1+Quadrant 4–1). In addition, activation of caspase-3, which is marked by cleavage of procaspase-3, is shown by Western blotting (Figure 7C), which corroborated with results obtained with fluorescence microscopy (Figure 6) and flow cytometry (Figure 7A and Figure 7B) analysis; caspase 3 is one of the key executioners of apoptosis. Thus, *P. indica* root aqueous extract at 0.5 mg/ml concentration effectively induced apoptosis/necrosis in HeLa cells.

### *P. indica* root aqueous extracts suppress cancer cell proliferation and migration through the phosphorylated-p53 and p21 pathways

**Figure 8:**
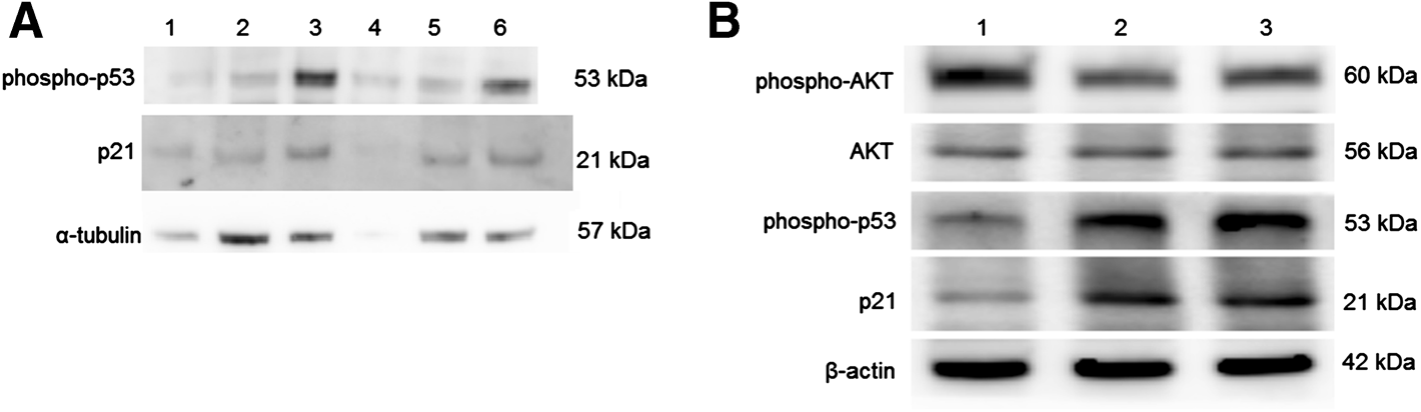
**Western blot analysis of phosphorylated-p53 and p21 proteins in cancer cells treated with *P. indica *leaf or root aqueous extract.** (**A**) Western blot of p21 and phosphorylated-p53 proteins of GBM8401 cells after *P. indica* extract treatment. Lane 1: untreated; lane 2: 3 days of 0.1 mg/ml *P. indica* root aqueous extract treatment; lane 3: 3 days of 1 mg/ml *P. indica* root aqueous extract treatment; lane 4: 5 days of 1 mg/ml *P. indica* root aqueous extract treatment; lane 5: 3 days of 0.1 mg/ml *P. indica* leaf aqueous extract treatment; lane 6: 3 days of 1 mg/ml *P. indica* leaf aqueous extract treatment. (**B**) Western blot of p21, phosphorylated-p53, AKT, and phosphorylated-AKT proteins of HeLa cells after *P. indica* extract treatment. Lane 1: untreated; lane 2: 2 days of 0.25 mg/ml *P. indica* root aqueous extract treatment; lane 3: 2 days of 0.5 mg/ml *P. indica* root aqueous extract treatment. Each experiment was done in triplicate.

To elucidate the underlying mechanism in the inhibition of cancer cell proliferation and migration by *P. indica* leaf or root aqueous extracts, Western blots of phosphorylated-p53 and p21 were examined. Phosphorylated-p53 and p21 were found to be induced in GBM8401 cells treated for 3 days with 1 mg/ml *P. indica* leaf (lane 6) and root (lane 3) aqueous extracts (Figure 8A). Phosphorylated-p53 and p21 were found to be induced and phosphorylated-AKT were found to be decreased in HeLa cells treated for 48 hours with 0.25 mg/ml and 0.5 mg/ml *P. indica* root aqueous extracts (Figure 8B).

## Discussion

This study examined the cell growth arrest, anti-proliferation, anti-migration, and pro-apoptosis properties of *Pluchea indica* leaf and root crude aqueous extracts in human brain malignant glioma cell line GBM8401, and human cervical carcinoma cell line HeLa. The underlying inhibitory mechanism of *P. indica* leaf and root crude aqueous extract was also investigated. It was detected that membrane lipid oxidation level in HeLa cells increased after treatment with *P. indica* crude aqueous extract. The data showed that *P. indica* leaf and root crude aqueous extracts were effective in suppressing the proliferation and migration of GBM8401 and HeLa cells. It was found that *P. indica* root crude aqueous extract inhibited focus formation of GBM8401 and HeLa cells, and promoted apoptosis in HeLa cells. The data indicated that phosphorylated-p53 and p21 were induced in *P. indica* root crude aqueous extract-treated GBM8401 and HeLa cells, and that phosphorylated-AKT was decreased in HeLa cells treated with *P. indica* root crude aqueous extract.

The medicinal properties of *P. indica* extracts have been studied previously. The methanol fraction of *P. indica* root extract was found to possess antioxidant activity [11]. The methanolic fraction of a chloroform extract of *P. indica* root was found to have anti-inflammatory activity [12]. Beta-sitosterol and stigmasterol isolated from *P. indica* root methanol extract was found to be able to neutralize viper and cobra venom and antagonize cobra venom-induced lethality, cardiotoxicity, neurotoxicity, and respiratory changes [13]. In contrast to previous studies, this is the first study to examine the effect of crude aqueous extracts of *P. indica* leaf and root on cancer cells. Tannins, saponins, flavonoids, phenols, and proanthocyanidins were detected in crude aqueous extracts of *P. indica* leaf and root, which showed that *P. indica* has reasonable anti-cancer potential because total phenolics, flavonoids, and tannins were shown to inhibit ATP-binding cassette transports in cancer cells [21], flavonoid intake was found to associate with a significant reduction in the risk of gastric cancer in women [22], saponins from *Radix astragali* were found to suppress colon cancer cell carcinogenic activity by reducing vascular endothelial growth factor [23], and proanthocyanidins from grape seeds inhibited pancreatic cancer cell growth and induced apoptosis [24].

Research into the anti-cancer potential of herbal extracts have been growing and expanding [6,25]. The compound, n-butylidenephthalide, isolated from the chloroform extract of *Angelica siensis* was found to upregulate the expression of p21 and p27 and increase apoptosis-associated proteins in DBTRG-05MG and RG2 cells and suppress the growth of subcutaneous rat and human brain tumors [26]. Nexrutine, a *Phellodendron amurense* bark methanol extract, was found to inhibit prostate cancer cell proliferation through modulation of AKT and cAMP-responsive element binding protein (CREB)-mediated signaling pathway, and that Nexrutine activates cyclin D1, which prevents the progression of prostate cancer [27]. In this study, it was observed that *P. indica* leaf and root aqueous extracts inhibited GBM8401 malignant glioma cells and HeLa cervical carcinoma cell growth, and migration. We found that the crude aqueous extract *P. indica* root suppressed focus formation of GBM8401 and HeLa cells.

The p53 tumor-suppressor gene does not function properly in most human cancers [28]. In brain cancers, p53 can be inactivated through amino acid-changing mutation in the DNA-binding domain and/or deletion of the p14^ARF^ gene, and in cervical cancers, p53 is inactivated through viral infection [29,30]. Phosphorylation of p53 by kinases, caused by DNA damage, leads to p53 activation [31]. Phosphorylation of p53 can also occur in response to oxidative stress through the platelet-derived growth factor β receptor (PDGFβ)-mediated ataxia telangiectasia mutated (ATM) kinase activation or direct ATM activation by oxidative stress [32,33]. One of the first consequences of p53 activation is cell cycle arrest through the p53-dependent expression of p21^WAF1/CIP1^, an inhibitor of cyclin-dependent kinases (CDKs) [34]. Upregulation of p53 and p21 were found to be induced by lipid peroxidation in previous studies: lipid peroxidation increase was associated with p53 mRNA increase in a rat model [35], lipid peroxidation product from increased ferrous iron level in lysosomal compartment triggered upregulation of p53 [36], lipid peroxidation product sensitizes cells to UV-induced killing by inhibiting nucleotide excision repair and forming a peroxide-DNA adduct at codon 249 of the p53 gene [37], and that hydrogen peroxide-induced lipid peroxide production increased p21 expression [38]. In the present study, it was observed that *P. indica* root crude aqueous extracts caused an increase in membrane lipid oxidation, and induced phosphorylated-p53 and p21expression in GBM8401 and HeLa cells.

The AKT/PKB (protein kinase B) kinases play important roles in signaling pathways that regulate cellular processes controlling cell proliferation, survival, and genome stability [39]. Hyperactivation of the AKT pathway was implicated in many types of human cancer and dominantly inherited cancer syndrome. AKT phosphorylates and inactivates the pro-apoptotic factors BAD and procaspase-9 [39]. In a pro-cell cycle progression mechanism involving p53, AKT promotes the phosphorylation and translocation of Mdm2 into the nucleus, where it downregulates p53, which antagonizes p53-mediated cell cycle checkpoints [40]. AKT directly antagonizes the function of the cell cycle inhibitors p21^WAF1^ and p27^Kip1^ by phosphorylating a site located near the nuclear localization signal to induce cytoplasmic retention of these cell cycle inhibitors [41]. Some investigations have shown elevated AKT activity to be highly prevalent in high grade, late stage and/or metastatic tumors, and several reports have linked AKT activation with reduced patient survival or tumor radio-resistance [42,43]. In the present study, *P. indica* root crude aqueous extract treatment lowered the expression of activated phosphorylated-AKT, without affecting the expression of non-phosphorylated AKT, and induced the expression of phosphorylated-p53 and p21 in HeLa cells. Therefore, the pro-apoptotic and anti-proliferation properties of the crude aqueous extract of *P. indica* root might be attributed to its induction of phosphorylated-p53 and p21 through the downregulation of activated phosphorylated-AKT, which causes cell cycle arrest and initiation of apoptosis leading to cancer cell death.

The active components in crude aqueous extracts of *P. indica* root may be good candidates for further investigation into the search for effective anti-cancer and synergistic drugs. It is also important to note that aqueous extracts of herbal substance was previously found to be less toxic than ethanol extracts of herbal material; the LD_50_ of aqueous and ethanol extracts were 12.30 g/kg and 6.15 g/kg, respectively [44]. We found that *P. indica* leaf and root crude aqueous extracts possess pro-oxidant, anti-proliferation, and anti-migration properties in GBM8401 malignant glioma cells and HeLa cervical carcinoma cells, and that *P. indica* root crude aqueous extract inhibited focus formation of these cancer cells. We also found that phosphorylated-p53 and p21 are induced in GBM8401 and HeLa cells after *P. indica* root crude aqueous extracts treatments. As keenly pointed out by a review, the challenge in CAM is to avoid contaminated products and those that may interact with prescription pharmaceuticals [6]. Further examination into the underlying anti-cancer mechanism and pharmacodynamic and pharmacokinetic interactions with current cancer drugs should be carried out to ensure effective clinical application and usage of *P. indica* leaf or root crude aqueous extract.

## Conclusions

In conclusion, the results of this study suggest that *P. indica* crude aqueous extracts has a promising anti-proliferative and anti-migratory effect on GBM8401 malignant glioma cells and HeLa cervical carcinoma cells. In addition, it was found that *P. indica* crude aqueous extracts induced critical tumor suppressor molecules, phosphorylated-p53 and p21, for cell cycle arrest and apoptosis, and downregulated an important survival signaling molecule, phosphorylated-AKT. Further investigations are needed to determine the entire anti-cancer molecular mechanism of *P. indica* aqueous extracts.

## Competing interests

The authors declare that they have no competing interests.

## Authors’ contributions

All authors contributed equally in data acquisition and interpretation of the manuscript. All the authors read and approved the final version of the manuscript.

## Pre-publication history

The pre-publication history for this paper can be accessed here:

http://www.biomedcentral.com/1472-6882/12/265/prepub
